# Verification of watershed vegetation restoration policies, arid China

**DOI:** 10.1038/srep30740

**Published:** 2016-07-29

**Authors:** Chengqi Zhang, Yu Li

**Affiliations:** 1 Key Laboratory of Western China’s Environmental Systems (Ministry of Education), College of Earth and Environmental Sciences, Center for Hydrologic Cycle and Water Resources in Arid Region, Lanzhou University, Lanzhou, 730000, China

## Abstract

Verification of restoration policies that have been implemented is of significance to simultaneously reduce global environmental risks while also meeting economic development goals. This paper proposed a novel method according to the idea of multiple time scales to verify ecological restoration policies in the Shiyang River drainage basin, arid China. We integrated modern pollen transport characteristics of the entire basin and pollen records from 8 Holocene sedimentary sections, and quantitatively reconstructed the millennial-scale changes of watershed vegetation zones by defining a new pollen-precipitation index. Meanwhile, Empirical Orthogonal Function method was used to quantitatively analyze spatial and temporal variations of Normalized Difference Vegetation Index in summer (June to August) of 2000–2014. By contrasting the vegetation changes that mainly controlled by millennial-scale natural ecological evolution with that under conditions of modern ecological restoration measures, we found that vegetation changes of the entire Shiyang River drainage basin are synchronous in both two time scales, and the current ecological restoration policies met the requirements of long-term restoration objectives and showed promising early results on ecological environmental restoration. Our findings present an innovative method to verify river ecological restoration policies, and also provide the scientific basis to propose future emphasizes of ecological restoration strategies.

Vegetation reconstruction is an effective approach for ecosystem restoration^1^,^2^. Well understanding of natural vegetation evolution process plays an important role in watershed ecosystem restoration, and various ecological restoration techniques have been widely used^3^,^4^,^5^. However, most of the restoration projects are considered unsuccessful since the environmental change is always asynchronous in different parts of a basin^6^,^7^. Verification of restoration policies and precise assessments of restoration strategies is of significance to simultaneously reduce global environmental risks while also meeting economic development goals^8^,^9^,^10^. Although many studies have been conducted on assessing basin-wide restoration strategies by data simulating or observing indicators of biodiversity^11^,^12^,^13^, there is not yet a widely accepted method to verify ecological restoration policies.

In this study, we proposed a novel method based on the idea of multiple time scales to verify ecological restoration policies in the Shiyang River drainage basin, where the Chinese government has implemented a series of ecological restoration policies, in which vegetation reconstruction is an essential aspect^14^,^15^. We synthesized modern pollen transport characteristics of the entire basin and pollen records from 8 Holocene sedimentary sections to quantitatively reconstruct the millennial-scale changes of watershed vegetation zones. Meanwhile, EOF (Empirical Orthogonal Function) method was used to quantitatively analyze spatial and temporal variations of NDVI (Normalized Difference Vegetation Index) in summer (June to August) of 2000–2014. In order to examine whether vegetation changes of various locations are synchronous in different time scales and study the availability of the current ecological restoration policies, vegetation changes, which were mainly controlled by millennial-scale natural ecological evolution, were compared with that under conditions of modern ecological restoration measures. In addition, we also elucidated the requirements of long-term restoration objectives. Our findings will address the future emphasizes of ecological restoration policies.

## Regional Setting

**Figure 1:**
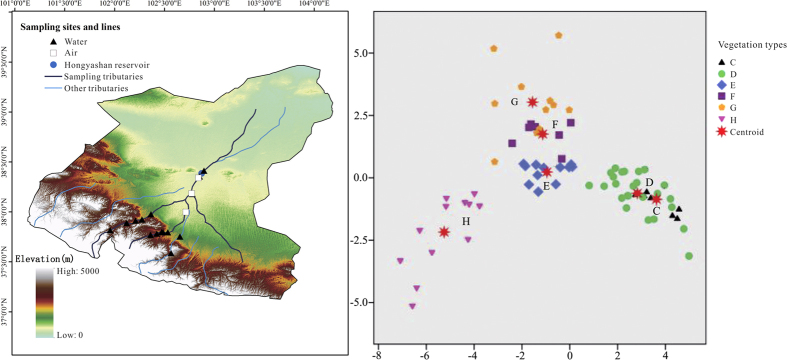
The elevations and modern pollen sample sites of the Shiyang River drainage basin (left) and locations of the results of the discriminant analysis along discriminant functions 1 and 2 (right). The modern pollen samples include 76 surface modern pollen samples, 28 lacustrine sediment samples, 17 water samples and 8 air samples. The letters in legends represent different vegetation types: C, subalpine scrub vegetation. D, forest vegetation. E, steppe vegetation. F, desert steppe vegetation. G, desert vegetation. H, sand desert vegetation. (Software: ArcGIS 10.0, SPSS Statistics 19, and CorelDRAW X6).

The Shiyang River, which originates from the Qilian Mountains and flows northeast toward the Gobi Desert, is located in the northeast of the Qinghai-Tibet Plateau, the eastern Hexi Corridor, arid China (Fig. 1). Climate of the Shiyang River drainage basin is mainly affected by the Asian monsoon system and the mid-latitude westerly winds^16^. The length of the drainage path is about 300 km, and the catchment area of the Shiyang River is about 41600 km^2^, which is roughly at geographical coordinates of 100°57′–104°57′E, 37°02′–39°17′N. The Shiyang River drainage basin, which is one of three major rivers in arid northwest China, is the region where the ecological environment problems in arid areas are most prominent^14^.

## Materials and Methods

The Shiyang River drainage basin is at a transition area between the Qinghai-Tibet Plateau, the Gobi Desert and monsoonal China, according to geographical divisions of China. The piedmont of the Qilian Mountains is widely covered by the Quaternary eolian sediments. The alluvial plain in the middle reaches are formed by the Quaternary unconsolidated lacustrine and alluvial sediments. And the terminal area of the drainage is a tectonic rift basin, belonging to the Qilian Mountains piedmont fault basin. Pollen records from 8 Holocene sedimentary sections (Supplementary Figure 1–7, Supplementary Table 1, 2) and 129 modern pollen samples (Supplementary Table 3) were collected to conduct a comparative study^17^,^18^,^19^,^20^,^21^,^22^,^23^,^24^,^25^,^26^. The sample treatment methods have been reported^18^,^19^,^20^,^21^,^22^,^23^,^27^,^28^ and numerical analyses for modern pollen data are as follows. The modern pollen data were calibrated since samples from the 8 Holocene sedimentary sections are lacustrine sediments while modern pollen samples are mainly from surface soil. Discriminant analysis was used to classify the modern pollen spectra into six pollen assemblages corresponding to the six vegetation zones. Hierarchical cluster analysis (HCA) and multidimensional scaling method (MDS) were used to divide the main pollen taxa into several groups representing different indicative significance of environment.

One series of 16-day NDVI data from Moderate Resolution Imaging Spectroradiometer (MODIS) sensor at spatial resolutions of 1000 m, which were downloaded from the Distribution Active Archive Center (DAAC) at NASA Goddard Space Flight Center (GSFC) for the summer (June to August) of 2000–2014, were quantitatively analyzed using EOF method to evaluate restoration effects of “The Shiyang River Basin (SRB) Comprehensive Restoration Plan”, which covered the entire Shiyang River basin, and officially implemented by the Chinese government since 2002 while some preparatory and experimental work started around 2000 15.

## Results

### Numerical analyses for modern pollen data

The results of the discriminant analysis confirm that the six vegetation regions have distinctive palynological signatures as represented by the six pollen assemblages (Fig. 1), and a comparison of the predicted group (pollen zone) memberships with the *a priori* groups shows that 86.7% of the samples are correctly classified (Supplementary Table 4, 5). Two major groupings are indicated by HCA and the palynological identification of these groups may provide a useful basis for palaeoenvironmental reconstruction (Supplementary Figure 8). The MDS procedure suggests two dimensions for the final solution (Supplementary Figure 9). The first dimension probably reflects the precipitation gradient while the second dimension reflects the temperature gradient. In addition, a gradient of decreasing elevation can be traced diagonally from the top left to the low right corner, as indicated by the modern geographic distribution of the six vegetation regions in the Shiyang River drainage basin.

Based on these numerical analyses and pollen transport characteristics, we divided the main pollen taxa into six pollen-climate groups (Supplementary Table 6), and propose a new pollen-precipitation index P which was calculated according to the ratios between groups of pollen taxa that represent different precipitation conditions in the study region: P = (G4 + G6)/(G1 + G2), in which G1, including *Artemisia*, Chenopodiaceae and Gramineae, and G2, including *Nitraria* and *Ephedra*, are representative of the dry climate, while G4, including *Picea*, and G6, including Rhamnaceae and *Pinus* represent the humid conditions. The mathematical relationship between the elevation E and modern precipitation R − P is as follows (Supplementary Dataset 1): R − P = 0.162^*^E−102.310 (R^2^ = 0.979, sig = 0.011), by using data gathered from meteorological stations. An optimum regression equation of P and R − P (Supplementary Table 7), which represents the real precipitation, is established: R − P = 76.242*P^0.441 (R^2^ = 0.949, sig = 0.005).

### Millennial-scale vegetation changes in the Shiyang River drainage basin

**Figure 2:**
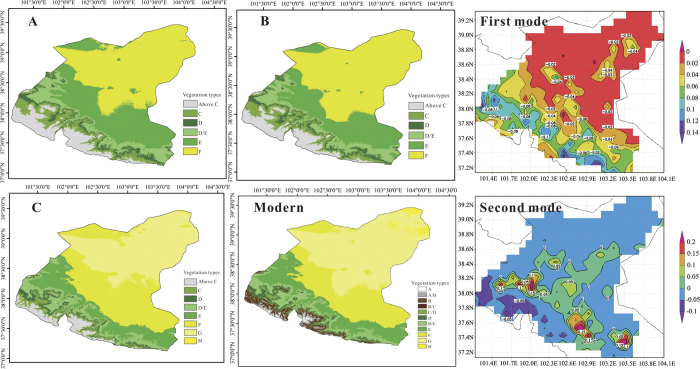
The vegetation types of the Shiyang River drainage basin in ~10000–~7000 cal a BP (**A**), ~7000–~3500 cal a BP (B), ~3500–~0 cal a BP (C) and modern times, and the first two EOF modes of NDVI in summer (June to August) of 2000–2014. The letters in legends represent different vegetation types: **A**, alpine cushion vegetation. B, alpine meadow vegetation. C, subalpine scrub vegetation. D, forest vegetation. E, steppe vegetation. F, desert steppe vegetation. G, desert vegetation. H, sand desert vegetation. Legends with “/” represent transition zones of vegetation types before and after “/”. (Software: ArcGIS 10.0, GrADS 2.0, and CorelDRAW X6).

The Holocene was generally divided into three time periods: A(~10000–~7000 cal a BP), B(~7000–~3500 cal a BP) and C(~3500–~0 cal a BP) (Supplementary Table 8). Based on the assumption that the surface sediments of each sedimentary sections are modern sediments, we calculated the pollen-precipitation index P’ of HS section, SJC section, QTH01 section, QTH02 section and QTL–03 section respectively during Holocene. The index of HS section, which lacks modem sediments, was calibrated using pollen data from section QTH01 and section QTH02 at 5000 cal a BP and 7000 cal a BP. A set of data, including magnetic susceptibility, carbonate content and total organic carbon content from HX section and pollen records from SKJ section and JTL section, was used to verify the calculated results. Ultimately, the quantitative reconstruction of millennial-scale vegetation changes in the Shiyang river drainage basin shows a clear and definite result (Fig. 2, Detailed calculation will be reported later). During period A, Subalpine scrub, forest, steppe and desert steppe were distributed at the elevation ranges of 2700~3100 m, 2500~2700 m, 1500~2100 m and below 1500 m. As the result of an intensifying drying trend, the elevation ranges of each vegetation zones shifted up about 50 m and 400 m during period B and period C, and vegetation types at the northeastern margin of Shiyang River drainage basin turned into sand desert during period C.

### Spatiotemporal analyses of modern summer NDVI

The first three EOF modes explain respectively 27.1%, 26.3% and 9.4% of the total variance (Supplementary Figure 10). The first mode reflects the impact of climate change since it is negative almost in the whole basin and its absolute values decreases with decreasing elevation, while vegetation changes controlled by climate change are synchronous in the whole basin. The first temporal EOF has maximum absolute value in 2002. The second mode reflects the characteristics of vegetation changes which were mainly influenced by human activities in the surrounding settlements, and its associated temporal EOF shows positive-negative shifts in every July or early August. The third mode may be interpreted as primarily characterizing vegetation changes affected by the Shiyang River as the variation of its associated temporal EOF is consistent with that of annual runoff of the Shiyang River.

The first EOF mode shows that the responses of vegetation to climate change in different parts of the Shiyang river drainage basin have consistency even though the responsiveness various as showed in the second mode (Fig. 2). Millennial-scale vegetation changes based on the pollen data from different locations also imply that the tendency of environmental change in the entire basin is same. The results of millennial-scale vegetation reconstruction and the first EOF mode suggest that vegetation changes of the Shiyang river drainage basin are both synchronous in these two time scales.

## Discussion

Unlike the general idea that environmental change in different locations of a basin is asynchronous, it is quite evident that vegetation changes of the entire Shiyang River drainage basin are synchronous whether in Holocene or modern times. Therefore, it is suitable to conduct a comparative study between multiple time scales and to verify ecological restoration policies in the Shiyang River drainage basin. The Chinese government have implemented “The Shiyang River Basin (SRB) Comprehensive Restoration Plan” (the Plan), whose restoration strategies could be summarized as follows: to protect the upstream forests which are the water supply and conserving area of the basin, adjust industrial structure especially in agriculture and construct a water-saving society in middle reaches, and restore groundwater levels and control desertification around the terminal lake. Meanwhile, obvious differences of responsiveness in various locations are shown in the second EOF mode and the distribution of values is mainly influenced by human activities in the surrounding settlements, which means the Plan varies in effectiveness depending on where it is applied. According to spatiotemporal analysis of summer NDVI in the Shiyang River drainage basin, we found that the Plan played an active role in upstream vegetation restoration, and also had significant effects on revegetation and living conditions improvement in oases of lower Shiyang River. However, the focus on enforcing the Plan in midstream needs to be increased as there are significantly high values at human settlements in the second EOF mode. Above all, we found that the current ecological restoration policies met the requirements of long-term restoration objectives and showed promising early results on ecological environmental restoration along the river basin.

The recognition of the ecological and social importance of watershed ecosystem has resulted in a massive increase in efforts and research of river basin restoration, which even has become a multibillion dollar industry worldwide. However, the scientific basis for watershed restoration remains uncertain and few restoration projects are considered successful^29^. To further the science of watershed restoration and achieve global sustainability, there is a need for restoration policies to be effectively verified to see if restoration objectives meet natural evolution process and restoration strategies are valid. The importance of understanding natural patterns and processes has been noticed^30^, and it is essential for effective restoration in a catchment area to understand how to determine the long-term goals of restoration efforts and identify restoration priorities since restoration objectives could vary broadly. There are also efforts as to what constitutes successful restoration^31^, but still lacking studies of fact verification for current ecological restoration policies in a catchment area as a whole. This research has shown that the novel method based on multiple time scales proves that the current ecological restoration policies met the requirements of long-term restoration objectives and showed promising early results on ecological environmental restoration along the river basin. Our findings also provide the scientific basis to propose future emphasizes of ecological restoration strategies.

## Additional Information

**How to cite this article**: Zhang, C. and Li, Y. Verification of watershed vegetation restoration policies, arid China. *Sci. Rep*. **6**, 30740; doi: 10.1038/srep30740 (2016).

## Supplementary Material

Supplementary Information

Supplementary Information
