# Supplementary material for: Verification of watershed vegetation restoration policies, arid China
Source: Sci Rep. 2016 Jul 29;6:30740. doi: 10.1038/srep30740 (PMC4965823; doi:10.1038/srep30740)
Supplement: Supplementary Information [file srep30740-s1.pdf]

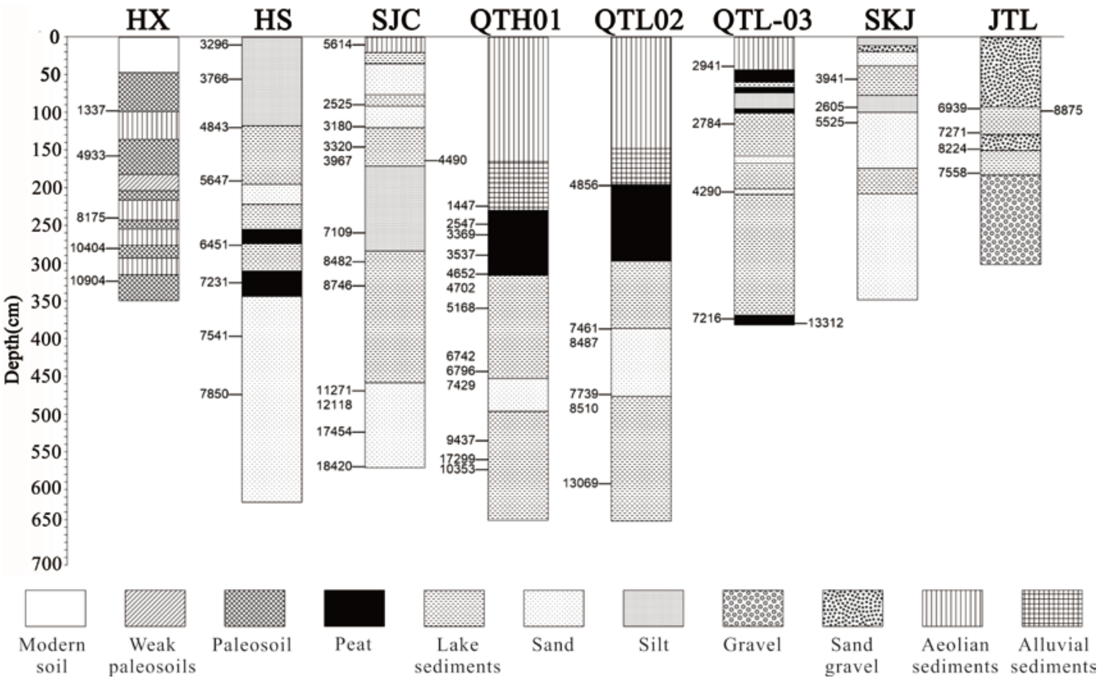

Supplementary Figure 1 Lithology and dates obtained from the HX, HS, SJC, QTH01, QTH02, QTL–03, SKJ and JTL sections. Using Calib5.01 software, all ^{14}C dates are calibrated to the calendar year.

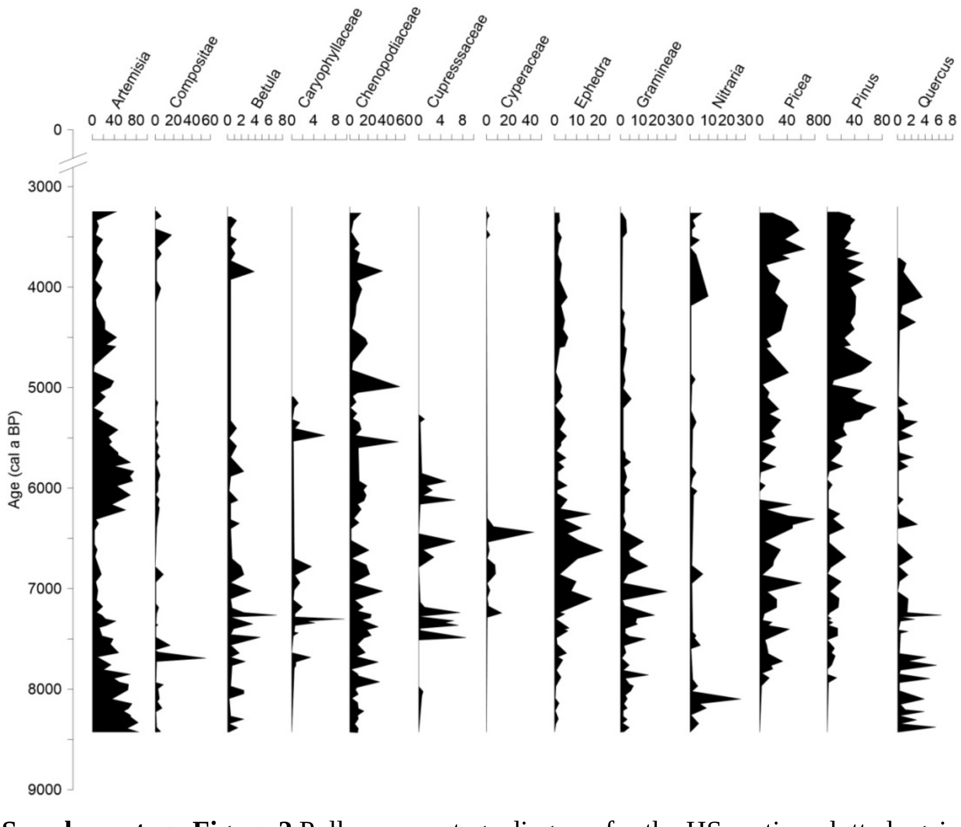

Supplementary Figure 2 Pollen percentage diagram for the HS section plotted against age.

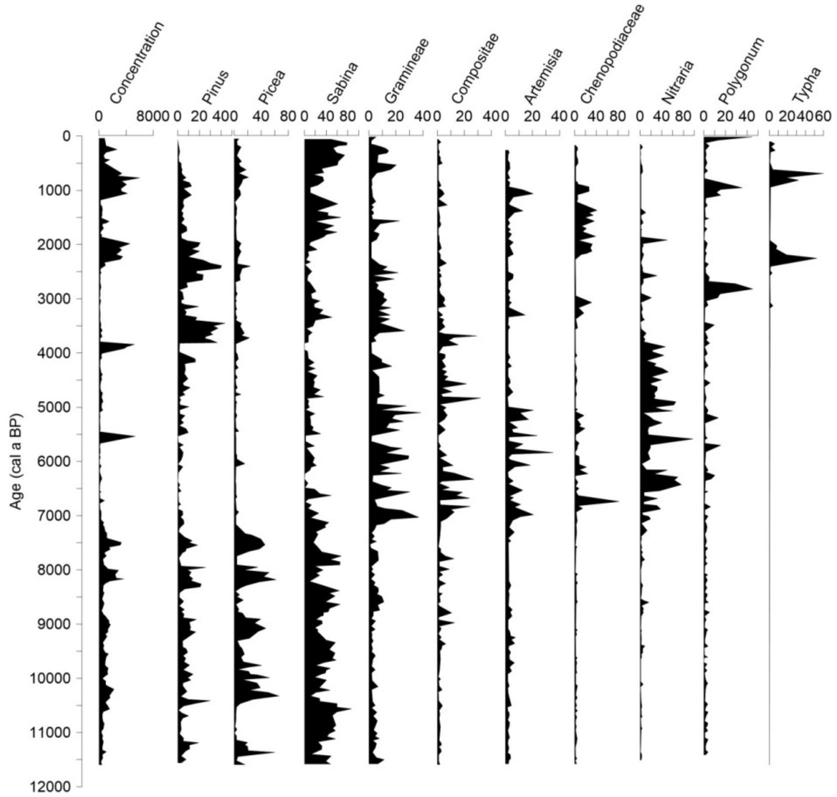

Supplementary Figure 3 Pollen percentage diagram for the SJC section plotted against age.

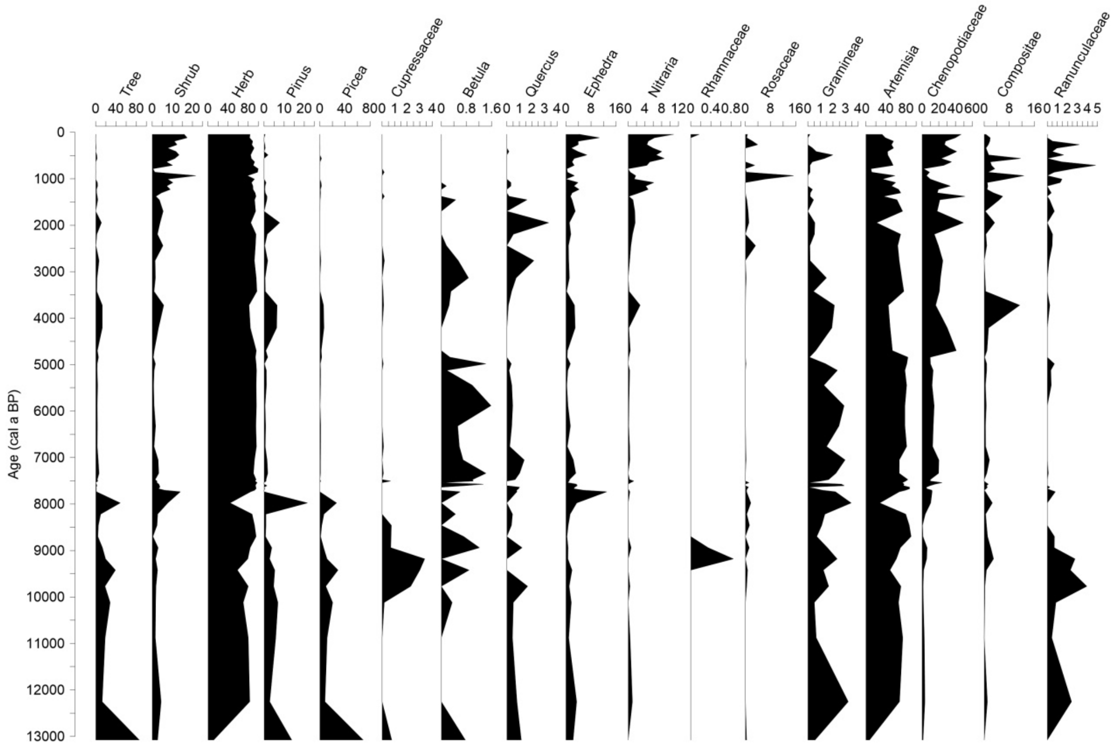

Supplementary Figure 4 Pollen percentage diagram for the QTH02 section plotted against age.

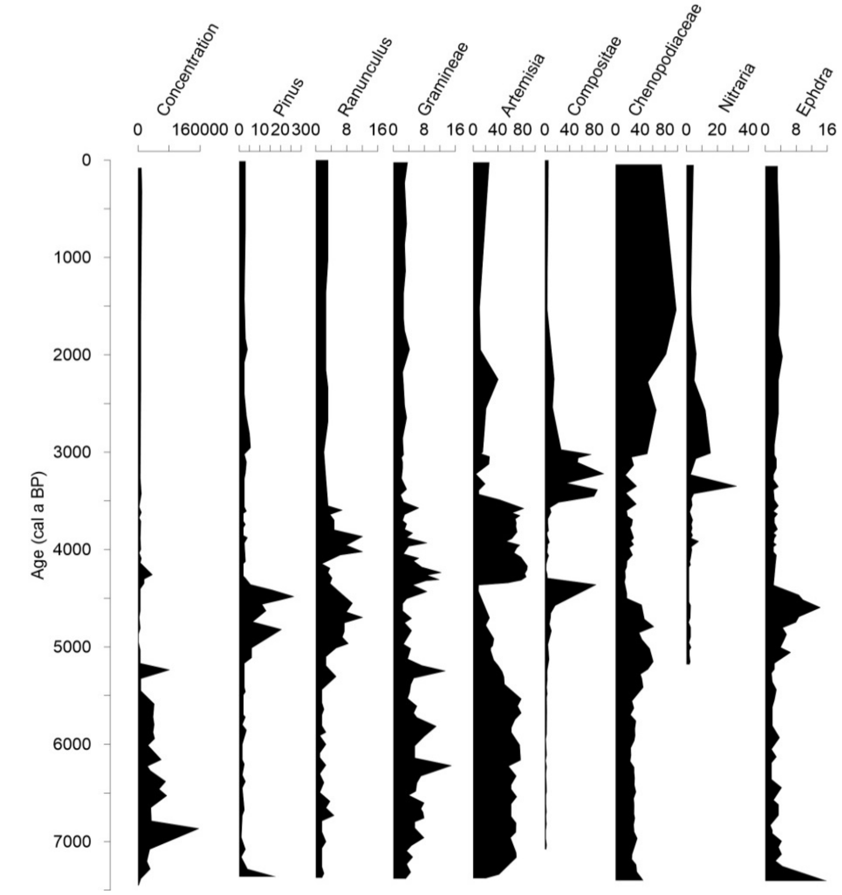

Supplementary Figure 5 Pollen percentage diagram for the QTL-03 section plotted against age.

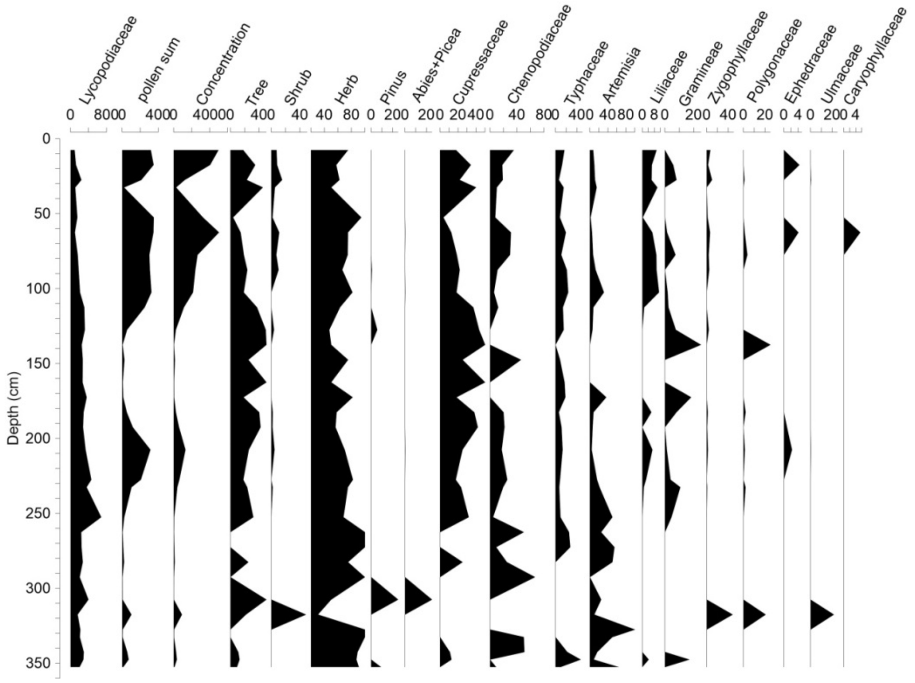

Supplementary Figure 6 Pollen percentage diagram for the SKJ section plotted against age.

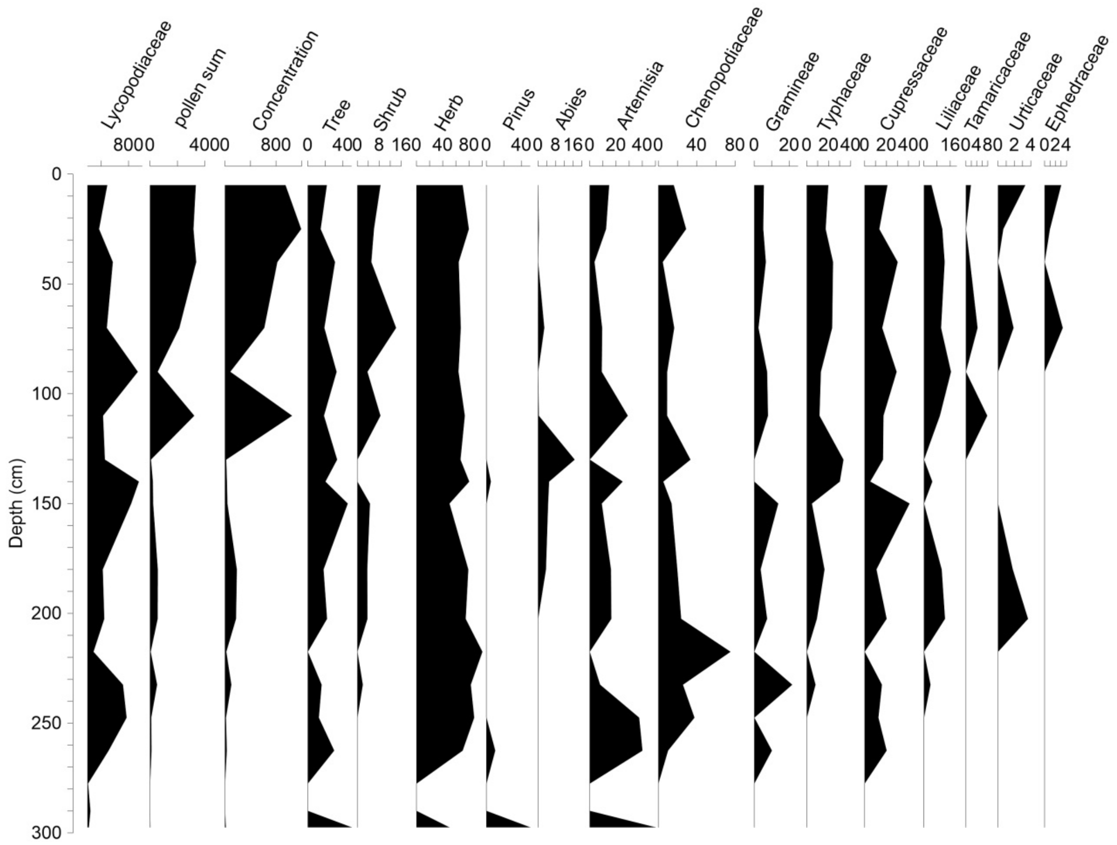

Supplementary Figure 7 Pollen percentage diagram for the JTL section plotted against age.

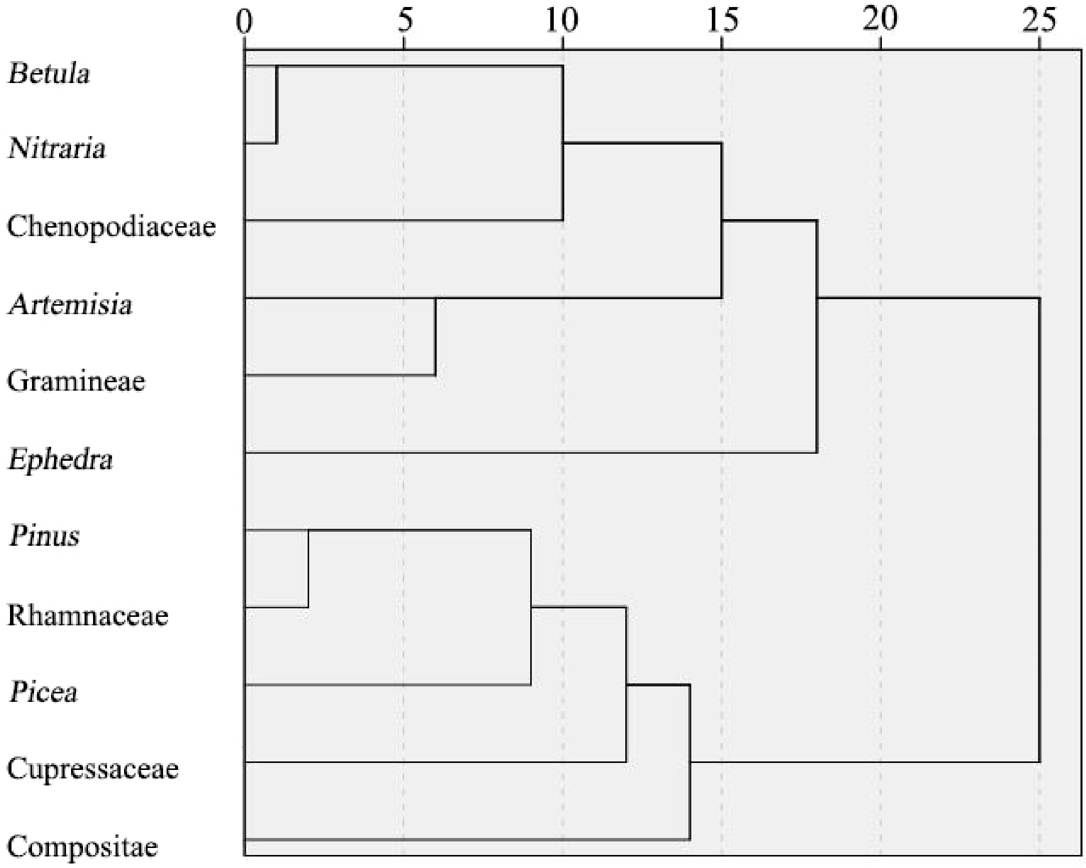

Supplementary Figure 8 Dendrogram of pollen taxon groups classified by hierarchical cluster analysis.

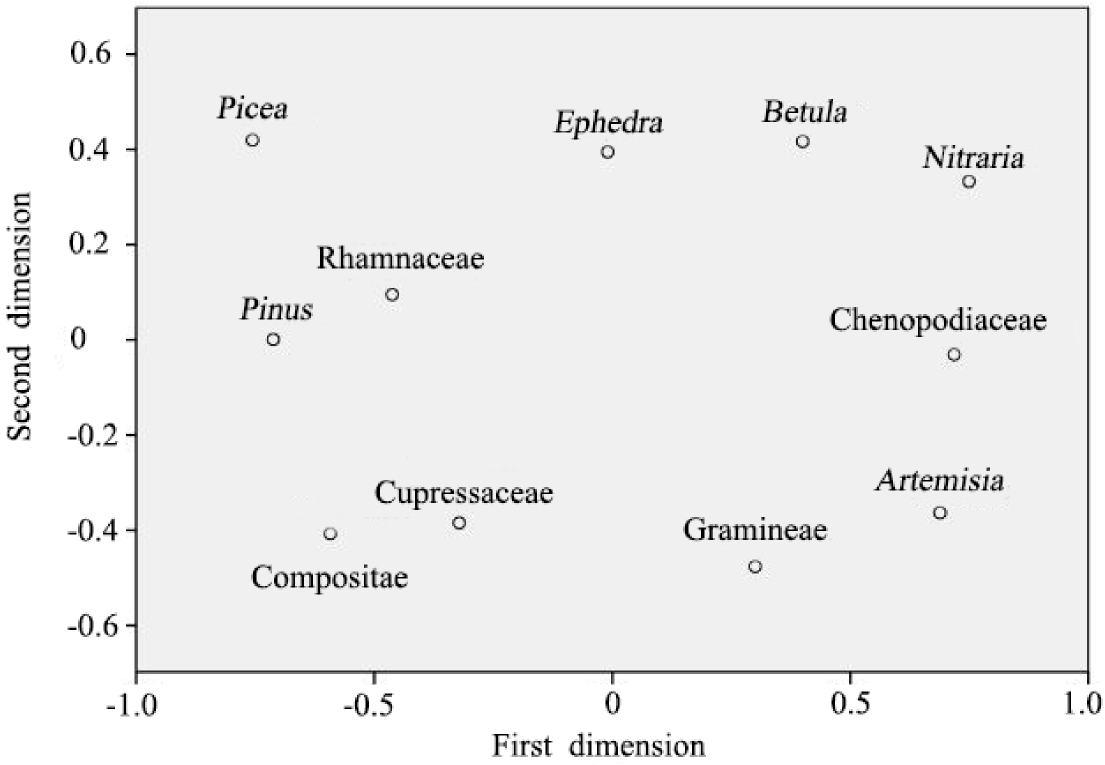

Supplementary Figure 9 Non-metric multidimensional scaling plot of pollen taxa in two-dimensional ordinal space, S-Stress=0.06906.

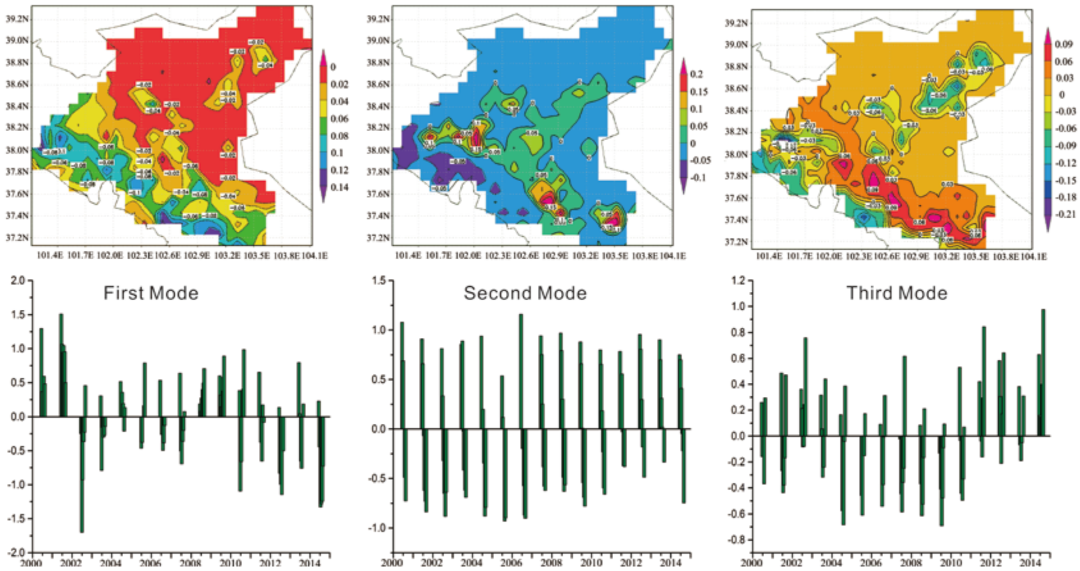

Supplementary Figure 10 The first three EOF modes of NDVI in summer (June to August) of 2000-2014. (Software: GrADS 2.0, Grapher 10 and CorelDRAW X6)

Supplementary Table1 The locations, elevations and depths of HX[20], HS[21], SJC[22-24], QTH01, QTH02, QTL–03[25], SKJ and JTL sections.

NO.	Sections	Location(lat,long)	Elevation (m)	Depth(m)	Reference
1	HX	37°30′N 102°24′E	2450	3.24	Wu et al.(1998)
2	HS	38°10′N 102°45′E	1460	6.20	Zhang et al.(2000)
3	SJC	39°00'N 103°20'E	1320	7.00	Chen et al.(2001, 2003, 2006)
4	QTH01	39°03'N 103°40'E	1309	7.50	This study
5	QTH02	39°03'N 103°40'E	1309	7.40	This study
6	QTL–03	39°04'N 103°36'E	1302	3.80	Zhao et al.(2008)
7	SKJ	39°00'N 103°52'E	1305	3.55	This study
8	JTL	39°09'N 104°08'E	1308	3.00	This study

Supplementary Table 2 ^{14}C dates for the HX[20], HS[21], SJC[22-24], QTH01, QTH02, QTL–03[25], SKJ and JTL sections.

Depth (m)	Dating materials	^{14}C age (a BP)	Calibrated ^{14}C age (2δ) (cal a BP)	Laboratory number/Reference
**HX**				
1.0	Organic matter	1420±50	1337(1304–1369)	Wu et al.(1998)
1.6	Organic matter	4320±70	4933(4850–5016)	Wu et al.(1998)
2.4	Organic matter	7350±150	8175(8032–8317)	Wu et al.(1998)
2.8	Organic matter	9200±115	10404(10276–10531)	Wu et al.(1998)
3.24	Organic matter	9560±120	10904(10716–11088)	Wu et al.(1998)
**HS**				
	Organic matter	/		Zhang et al.(2000)
0.09	Organic matter	/	3296(3202–3390)	Zhang et al.(2000)
0.57	Organic matter	/	3766(3650–3882)	Zhang et al.(2000)
1.20	Organic matter	/	4843(375–4933)	Zhang et al.(2000)
1.90	Organic matter	/	5647(5527–5767)	Zhang et al.(2000)
2.75	Organic matter	/	6451(6328–6574)	Zhang et al.(2000)
3.25	Organic matter	/	7231(7156–7360)	Zhang et al.(2000)
3.97	Organic matter(roots)	/	7541(7471–7611)	Zhang et al.(2000)
4.74	Organic matter(roots)	/	7850(7830–7870)	Zhang et al.(2000)
**XQ**				
0.45	Organic matter	3628±58	3946(3731–4144)	Zhao et al.(2008)
0.48	Pollen concentrates	5855±30	6678(6567–6745)	BA101249
1.62	Organic matter	5176±68	5937(5746–6177)	Zhao et al.(2008)
2.28	Organic matter	3758±65	4126(3926–4405)	Zhao et al.(2008)
3.51	Organic matter	21101±220	25220(24539–25862)	Zhao et al.(2008)
3.98	Pollen concentrates	22380±100	27063(26301–27716)	BA101248
4.29	Organic matter	18803±207	22431(21816–23290)	Zhao et al.(2008)
6.00	Organic matter	22158±189	26618(26052–27580)	Zhao et al.(2008)
6.03	Pollen concentrates	21000±120	25045(24575–25510)	BA101247
6.23	Organic matter	10400±80	12275(12029–12555)	Zhao et al.(2008)
7.42	Organic matter	12688±117	14992(14237–15572)	Zhao et al.(2008)
8.13	Pollen concentrates	15360±60	18619(18499–18792)	BA101246
8.27	Organic matter	11650±110	13512(13290–13753)	Zhao et al.(2008)
**SJC**				
0.10	Organic matter	4873±130	5614(5321–5902)	Chen et al.(2001,2003,2006)
0.90	Organic matter	2450±50	2525(2356–2708)	Chen et al.(2001,2003,2006)
1.20	Organic matter	3000±95	3180(2893–3393)	Chen et al.(2001,2003,2006)
1.45	Organic matter	3110±80	3320(3077–3548)	Chen et al.(2001,2003,2006)
1.45	Organic matter	3641±95	3967(3699–4235)	Chen et al.(2001,2003,2006)
1.65	Organic matter	4010±60	4490(4294–4804)	Chen et al.(2001,2003,2006)
2.60	Organic matter	6214±75	7109(6910–7274)	Chen et al.(2001,2003,2006)
3.00	Organic matter	7670±135	8482(8181–8969)	Chen et al.(2001,2003,2006)
3.30	Organic matter	7888±140	8746(8408–9086)	Chen et al.(2001,2003,2006)

4.70	Organic matter	9840±90	11271(10882–11692)	Chen et al.(2001,2003,2006)
4.70	Organic matter	10374±260	12118(11275–12680)	Chen et al.(2001,2003,2006)
5.25	Organic matter	14340±250	17454(16869–18023)	Chen et al.(2001,2003,2006)
5.40	Pollen concentrates	15900±140	19104(18798–19412)	Chen et al.(2001,2003,2006)
5.70	Organic matter	15262±450	18420(17473–19416)	Chen et al.(2001,2003,2006)
**QTH01**				
2.25	Organic matter	1550±60	1447(1316–1551)	LUG96–44
2.50	Organic matter	2470±90	2547(2351–2740)	LUG96–45
2.62	Shells	3140±40	3369(3263–3448)	BA05223
2.90	Organic matter	3300±90	3537(3356–3821)	LUG96–46
3.15	Organic matter	4130±110	4652(4298–4953)	LUG96–47
3.15	Shells	4160±40	4702(4571–4831)	BA05224
3.60	Organic matter	4530±80	5168(4881–5449)	LUG96–48
4.25	Organic matter	5960±65	6796(6652–6953)	LUG96–49
4.25	Shells	5920±40	6742(6658–6854)	BA05225
4.25	Pollen concentrates	6510±40	7429(7322–7494)	BA101234
5.37	Organic matter	8412±62	9437(9293–9530)	LUG02–25
5.61	Pollen concentrates	14220±50	17299(16989–17599)	BA101237
5.72	Organic matter	9183±60	10353(10234–10502)	LUG02–23
**QTH02**				
1.99	Pollen concentrates	4300±25	4856(4830–4958)	BA101254
3.88	Shells	6550±40	7461(7344–7563)	BA05222
3.88	Pollen concentrates	7705±35	8487(8413–8575)	BA101256
4.75	Shells	6910±40	7739(7671–7833)	BA05221
4.75	Pollen concentrates	7735±35	8510(8432–8587)	BA101257
5.91	Shells	11175±50	13069(12875–13241)	BA05218
**QTL–03**				
0.40	Organic matter	2835±35	2941(2857–3063)	Zhao et al.(2008)
1.18	Organic matter	2675±40	2784(2744–2853)	Zhao et al.(2008)
2.06	Organic matter	3860±35	4290(4156–4412)	Zhao et al.(2008)
3.74	Organic matter	6285±35	7216(7159–7289)	Zhao et al.(2008)
3.80	Organic matter	11445±50	13312(13180–13428)	Zhao et al.(2008)
**JTL**				
0.98	Organic matter	6071±80	6939(6744–7162)	LUG–03–08
1.00	Pollen concentrates	8000±40	8875(8663–9009)	BA101253
1.29	Organic matter	6350±114	7271(6987–7475)	LUG–03–07
1.50	Organic matter	7410±140	8224(7952–8454)	LUG–03–06
1.81	Organic matter	6688±100	7558(7419–7732)	LUG–03–05
**SKJ**				
0.58	Pollen concentrates	3630±25	3941(3866–4070)	BA101239
0.93	Organic matter	2541±57	2605(2366–2759)	Zhao et al.(2008)
1.14	Organic matter	4808±70	5525(5324–5659)	Zhao et al.(2008)

Supplementary Table 3 Pollen percentages of modern pollen assemblages in the Shiyang River drainage basin. The letters represent different vegetation types. C, subalpine scrub vegetation. D, forest vegetation. E, steppe vegetation. F, desert steppe vegetation. G, desert vegetation. H, sand desert vegetation.

Vegetation type	Sample type	*Artemisia*	*Betula*	Chenopodiaceae	Compositae	*Ephedra*	Gramineae	*Nitraria*	*Picea*	*Pinus*	Rhamnaceae	Cupressaceae
C	Surface soil	0.00	0.00	0.00	0.00	0.21	4.58	0.00	81.44	4.50	0.42	8.99
C	Surface soil	1.27	0.84	0.00	2.85	0.00	7.51	0.60	78.16	4.50	0.00	9.74
C	Surface soil	0.00	0.84	0.00	2.39	0.00	4.17	0.00	67.44	4.50	0.34	2.17
C	Surface soil	0.00	0.00	0.00	6.20	0.00	4.99	0.60	74.16	4.50	0.42	4.62
C	Surface soil	0.00	0.00	0.00	45.23	0.00	0.00	0.00	32.80	2.75	0.34	5.75
C	Surface soil	0.00	0.00	0.00	19.05	0.17	6.26	0.60	64.80	3.75	0.58	3.35
C	Surface soil	0.00	0.00	0.00	15.24	0.00	6.26	0.60	57.44	3.75	0.50	8.96
C	Surface soil	0.00	5.20	3.72	3.34	0.34	12.09	3.00	54.16	2.49	1.16	2.36
D	Surface soil	2.97	0.00	0.00	10.47	0.00	4.99	0.00	46.16	3.75	0.34	1.14
D	Surface soil	0.00	0.00	0.00	5.73	0.15	4.17	0.60	68.16	4.25	1.00	3.2
D	Surface soil	0.00	0.00	0.00	6.66	0.00	2.09	0.00	65.44	3.50	0.34	1.38
D	Surface soil	0.00	3.87	0.00	9.05	0.00	2.93	0.72	76.80	2.75	0.34	1.32
D	Surface soil	0.00	0.00	0.00	5.24	0.00	2.93	0.60	77.44	1.74	0.50	3.94
D	Surface soil	0.00	0.00	0.00	21.90	0.00	2.09	0.00	56.80	2.00	0.00	0.19
D	Surface soil	0.00	0.00	0.00	5.24	0.29	2.49	0.72	56.80	4.76	1.16	6.13
D	Surface soil	0.00	0.00	0.00	3.81	0.00	6.26	0.00	59.44	1.50	0.42	4.81
D	Surface soil	0.00	1.51	0.00	2.85	0.29	2.93	0.84	74.80	2.00	0.83	6.38
D	Surface soil	0.00	0.00	7.47	1.92	0.23	2.09	0.96	55.44	1.24	0.58	4.32
D	Surface soil	2.53	1.86	0.00	1.92	0.00	2.93	0.00	68.80	2.00	0.75	1.49
D	Surface soil	0.00	1.02	0.00	0.96	0.13	2.49	0.48	73.44	2.25	0.42	1.43
D	Surface soil	0.00	0.00	0.00	1.92	0.00	3.33	0.00	68.16	1.50	0.50	0.42
D	Surface soil	0.00	1.51	0.00	3.34	0.19	3.33	0.00	77.44	1.50	0.00	3.83
D	Surface soil	0.00	1.19	0.00	1.92	0.00	1.25	1.08	88.16	0.99	0.34	0.3
D	Surface soil	0.00	1.02	0.00	0.00	0.00	1.25	0.48	96.80	1.24	0.42	0
D	Surface soil	0.00	2.35	0.00	1.92	0.21	1.25	0.60	82.16	0.99	0.75	3.26
D	Surface soil	1.27	10.76	2.24	2.39	0.00	3.74	0.72	68.80	3.50	0.92	3.35
D	Surface soil	0.00	2.01	0.00	2.39	0.00	2.09	0.60	89.44	0.99	0.42	0.3
D	Surface soil	0.00	10.26	0.00	0.96	0.29	3.33	0.72	81.44	1.74	0.34	2.76
D	Surface soil	0.00	8.58	0.00	1.43	0.00	2.49	0.60	79.44	2.00	0.42	1.32
D	Surface soil	6.89	0.00	5.59	7.51	0.00	9.30	3.10	70.20	1.79	1.81	4.75
D	Surface soil	4.89	0.00	3.55	10.15	0.00	8.79	3.10	68.93	2.80	2.31	3.74
D	Surface soil	10.57	2.13	7.62	6.19	0.00	12.64	3.45	48.12	1.79	4.65	0
D	Surface soil	6.89	0.00	3.55	6.19	0.00	14.29	2.30	63.76	3.01	2.84	0
D	Lacustrine sediment	0.00	0.33	1.01	3.04	0.00	4.27	0.00	78.46	3.55	3.68	4.78
E	Surface soil	0.00	2.85	0.00	6.20	0.08	3.74	0.96	78.80	2.75	0.42	1.86

E	Surface soil	0.00	2.35	2.60	8.09	0.15	5.83	0.60	76.16	1.50	0.50	2.05
E	Surface soil	0.00	1.34	0.00	9.05	0.29	5.83	0.60	72.80	3.24	0.00	3.01
E	Surface soil	0.00	1.19	0.00	7.16	0.00	9.16	1.32	70.16	3.00	0.34	0.36
E	Surface soil	0.00	2.53	36.64	0.00	0.15	17.91	0.84	34.80	1.24	0.50	0.3
E	Surface soil	0.00	2.35	2.24	24.75	0.00	14.99	0.84	44.16	1.74	0.34	0.74
E	Surface soil	8.11	2.35	6.11	6.60	0.00	8.79	2.30	52.68	1.79	8.50	0
E	Surface soil	0.00	3.05	3.03	7.51	0.00	0.00	2.70	70.20	0.00	2.07	0.77
E	Surface soil	0.00	5.19	6.58	11.02	0.00	5.50	3.45	52.01	3.40	2.84	1.04
E	Surface soil	8.94	2.84	22.30	4.42	0.00	4.37	2.30	55.91	1.79	2.58	1.04
E	Surface soil	8.52	0.00	6.58	12.79	0.00	8.79	2.70	58.52	2.41	1.54	8.27
E	Surface soil	8.11	1.89	12.69	17.21	0.00	4.37	2.70	60.47	3.01	0.00	4.26
E	Lacustrine sediment	0.00	0.17	0.76	0.00	0.00	1.15	0.59	83.07	0.00	0.74	1.46
E	Lacustrine sediment	2.17	0.00	1.52	0.00	0.00	2.30	1.47	54.92	0.00	1.26	3.56
E	Lacustrine sediment	0.00	0.59	1.78	0.00	0.00	1.53	0.00	64.64	0.00	0.74	3.45
E	Lacustrine sediment	0.69	0.00	1.78	0.00	0.00	1.92	0.00	62.08	0.00	0.95	3.36
E	River water	6.30	1.60	4.70	3.10	1.60	4.70	4.70	17.20	12.50	1.60	17.1
E	River water	2.70	18.70	4.40	1.80	0.00	16.90	0.90	30.70	4.40	0.90	0.9
F	Surface soil	0.00	1.86	0.00	5.73	0.00	27.48	0.00	52.80	4.25	0.00	0
F	Surface soil	3.80	3.37	2.24	9.54	0.00	20.00	0.96	50.80	5.50	0.50	1.13
F	Surface soil	0.00	5.19	40.52	4.87	0.00	3.80	2.30	47.45	2.41	1.54	3.5
F	Surface soil	14.58	0.00	6.58	10.15	0.00	26.93	2.70	38.32	3.01	2.84	5.24
F	Surface soil	4.89	2.35	2.04	14.11	0.00	7.14	2.70	62.42	4.60	1.54	3.51
F	Surface soil	22.31	1.89	9.61	6.60	0.00	11.51	2.70	58.52	4.00	0.00	1.28
F	Surface soil	17.42	0.00	8.09	16.34	0.00	15.36	3.45	40.34	5.79	0.00	2.05
F	Lacustrine sediment	1.14	1.00	3.30	0.00	0.00	2.68	1.18	63.11	0.00	0.00	5.01
F	Lacustrine sediment	0.69	0.00	0.76	0.00	0.00	1.53	1.18	83.07	0.00	0.00	1.56
F	Lacustrine sediment	3.20	0.33	18.30	0.00	0.00	2.68	0.00	51.33	0.00	1.05	0.95
F	Lacustrine sediment	1.26	0.00	9.67	0.00	0.00	2.30	1.18	69.25	0.00	0.00	3.52
F	Lacustrine sediment	1.49	0.00	1.27	0.00	0.00	10.03	2.06	64.13	0.00	0.00	5.03
F	Lacustrine sediment	1.37	1.17	0.76	0.00	0.00	11.56	0.00	65.67	0.00	0.00	4.6
F	Lacustrine sediment	0.80	0.25	2.28	0.00	0.00	3.51	0.00	77.44	0.00	0.00	3.32
F	Lacustrine	1.60	0.84	8.66	0.00	0.00	14.63	1.18	60.04	0.00	0.32	0.32

	sediment											
F	River water	4.40	2.20	5.60	3.30	0.00	35.60	2.20	28.90	1.10	0.00	2.2
F	River water	6.90	17.20	3.40	0.00	0.00	3.40	0.00	17.20	1.70	3.40	10.3
F	River water	22.20	1.40	16.40	2.90	1.90	8.70	9.70	7.20	0.00	0.00	17.9
F	River water	4.20	2.80	4.20	2.80	0.00	13.90	1.40	41.70	5.60	1.40	1.4
F	River water	6.10	6.10	4.90	7.30	0.00	2.40	2.40	24.40	6.10	1.20	4.9
F	River water	0.00	0.70	2.00	3.00	0.30	28.90	0.00	50.50	5.30	0.00	0.3
F	River water	20.40	3.30	15.60	4.70	0.50	4.30	4.30	14.20	10.00	1.40	8.1
F	Air	3.21	1.55	10.86	1.38	0.86	22.24	4.14	2.41	1.03	0.52	2.59
F	Air	9.33	0.00	22.67	1.78	1.11	21.11	15.11	14.22	2.00	0.00	1.33
F	Air	2.84	0.00	9.63	1.75	0.88	35.01	7.66	31.07	1.75	0.00	1.75
F	Air	11.76	0.31	11.46	4.64	0.62	40.25	8.98	9.91	2.79	0.62	1.24
G	Surface soil	9.29	0.00	16.45	6.66	0.00	12.90	1.32	40.80	3.75	0.34	0
G	Surface soil	27.91	2.53	8.95	6.20	0.00	7.07	0.84	40.80	0.00	0.34	3.14
G	Surface soil	8.44	1.86	7.84	19.52	0.00	14.17	2.52	28.16	5.75	0.67	0.92
G	Surface soil	5.91	3.02	14.94	8.58	0.13	11.65	2.04	24.80	9.51	0.50	1.62
G	Surface soil	3.68	2.59	5.59	17.66	0.00	17.57	3.45	41.61	5.40	0.00	0
G	Surface soil	27.16	1.89	9.14	10.61	0.00	26.36	3.85	18.19	3.61	1.30	5.08
G	Surface soil	14.58	4.70	8.09	7.92	0.00	18.65	5.75	38.99	5.01	1.81	0
G	Surface soil	7.73	0.00	4.54	4.87	0.00	51.64	2.30	14.90	5.20	1.30	3.87
G	Surface soil	19.89	7.52	6.11	6.60	0.00	29.08	8.45	8.46	4.80	1.30	0
G	Surface soil	2.05	1.41	1.51	3.96	0.00	20.86	7.30	3.89	3.61	0.00	0
G	Surface soil	17.42	1.19	11.64	18.53	0.00	47.22	4.60	0.00	5.20	0.00	0
G	Lacustrine sediment	1.49	1.42	3.30	0.00	0.00	0.00	1.76	56.45	0.00	0.00	9.04
G	Lacustrine sediment	1.60	0.00	1.52	0.00	0.00	36.55	2.06	38.46	0.00	2.42	3.53
G	Lacustrine sediment	2.74	1.42	1.27	0.00	0.00	43.07	1.76	32.83	0.00	0.00	5.3
G	Lacustrine sediment	2.97	0.33	1.52	0.00	0.00	3.89	0.00	57.48	0.00	0.63	1.05
G	Lacustrine sediment	1.60	1.42	2.54	0.00	0.00	1.53	0.00	56.45	0.00	0.00	9.67
G	Lacustrine sediment	1.03	0.92	5.87	0.00	0.00	13.10	0.00	52.36	0.00	0.00	1.24
G	Lacustrine sediment	2.06	0.76	1.78	0.00	0.00	5.81	0.00	72.32	0.00	0.00	1.45
G	Lacustrine sediment	0.80	1.17	1.52	0.00	0.00	17.32	0.00	50.31	0.00	0.00	3.53
G	Lacustrine sediment	3.54	0.00	7.14	0.00	0.00	5.42	0.00	37.95	0.00	0.00	2.28
G	Lacustrine sediment	3.31	0.00	4.86	0.00	0.00	5.42	0.00	37.95	0.00	0.00	3.22

G	Lacustrine sediment	2.17	0.25	15.25	0.00	0.00	5.81	1.76	38.46	0.00	0.00	2.19
G	Lacustrine sediment	3.09	0.00	5.62	0.00	0.00	36.16	3.53	27.20	0.00	0.00	2.49
G	River water	18.60	4.50	11.20	4.10	1.20	13.20	0.80	28.50	2.50	0.00	2.5
G	River water	5.00	3.60	13.60	14.30	0.00	20.00	0.00	17.90	3.60	0.70	1.4
G	River water	20.60	0.60	13.50	8.80	0.00	11.10	4.10	8.20	5.30	0.60	10.6
G	River water	13.10	3.50	8.70	11.20	0.00	26.30	0.00	10.50	4.70	0.60	2.7
G	River water	18.20	4.50	4.50	9.10	0.00	22.70	0.00	4.50	0.00	0.00	13.6
G	River water	17.20	3.20	8.40	5.20	0.00	9.60	1.60	18.40	20.00	0.00	1.2
G	Air	11.02	0.85	12.71	0.85	6.78	15.25	3.39	1.69	0.85	0.00	1.69
H	Surface soil	0.00	0.00	4.07	6.19	0.00	5.50	23.85	5.84	5.01	0.00	0
H	Surface soil	28.37	1.89	6.11	6.60	0.00	36.79	14.60	7.11	7.01	1.04	0
H	Surface soil	29.58	1.89	6.58	4.87	0.00	38.44	13.85	4.50	7.20	0.00	0
H	Surface soil	8.52	0.00	11.17	7.92	0.00	25.79	1.94	24.03	4.00	1.04	13.21
H	Surface soil	3.26	0.00	4.54	10.61	0.00	40.65	11.15	0.00	2.00	1.04	0
H	Surface soil	0.00	0.00	1.04	30.46	0.00	1.64	3.85	0.00	1.40	0.00	3.51
H	Surface soil	7.31	0.00	8.62	16.75	0.00	43.93	7.30	1.95	5.20	0.00	1.5
H	Surface soil	3.68	2.59	10.13	3.10	0.00	28.00	9.25	3.22	8.00	0.00	0
H	Surface soil	27.95	0.95	6.58	10.61	0.00	29.65	5.40	0.00	6.00	0.00	4.53
H	Surface soil	16.63	0.95	13.68	6.19	0.00	28.57	13.10	1.28	4.00	0.00	0
H	Surface soil	6.10	6.59	7.10	6.19	0.00	14.80	25.00	3.22	3.79	0.00	2.52
H	Surface soil	12.99	0.00	19.27	15.02	0.00	20.29	20.79	3.89	3.61	0.00	7.26
H	Surface soil	10.95	0.00	14.20	23.81	0.00	20.29	6.95	3.89	5.01	0.00	0.51
H	Lacustrine sediment	0.80	0.51	1.78	0.00	0.00	3.89	2.06	48.70	0.00	0.00	1.45
H	Lacustrine sediment	1.60	1.33	8.91	0.00	0.00	3.07	8.24	37.44	0.00	0.21	0.83
H	Lacustrine sediment	1.49	1.33	5.36	0.00	0.00	3.07	9.41	46.65	0.00	0.95	1.03
H	River water	6.50	2.70	5.10	13.40	0.00	18.80	2.20	21.40	13.40	0.00	2.2
H	River water	14.90	2.10	16.30	12.80	2.40	15.60	8.70	9.00	4.10	0.00	1
H	Air	0.71	1.02	4.56	4.80	1.26	12.04	34.07	3.62	10.94	0.16	1.18
H	Air	2.57	1.20	7.11	5.65	0.86	16.44	30.39	2.91	4.71	0.51	1.63
H	Air	0.66	2.16	4.09	4.39	2.95	13.59	32.96	3.01	4.63	0.24	0.66

Supplementary Table 4 Standardized discriminant function coefficients used in the discriminant analysis.

Pollen taxa	Function				
	1	2	3	4	5
Betula	0.258	0.167	-0.029	0.136	-0.072
Picea	0.752	0.464	-0.032	0.113	0.473
Cupressaceae	0.204	-0.024	-0.046	-0.127	-0.171
Ephedra	0.248	-0.233	0.511	-0.642	0.010
Nitraria	-0.658	-0.640	0.303	0.267	0.018
Artemisia	-0.293	0.251	0.214	0.042	0.014
Chenopodiaceae	-0.102	0.298	0.209	0.249	0.882
Compositae	0.504	0.049	0.604	-0.262	0.131
Gramineae	0.256	0.882	0.553	0.517	-0.136
Pinus	0.611	-0.357	0.333	0.496	0.137
Rhamnaceae	0.133	-0.420	-0.623	0.075	-0.287
Percentage of Variance (%)	66.2	18.9	10.3	3.9	0.7
Cumulative percentage of variance (%)	66.2	85.1	95.5	99.3	100.0

Supplementary Table 5 Classification results of the modern pollen samples by discriminant analysis. 86.7% of the original grouped cases were correctly classified.

Vegetation	Predicted group membership					
type	C	D	E	F	G	H
C	5(62.5%)	3(37.5%)	0	0	0	0
D	0	25(100%)	0	0	0	0
E	0	0	12(100%)	0	0	0
F	0	0	1(14.3%)	5(71.4%)	1(14.3%)	0
G	0	0	0	5(45.5)	6(54.5%)	0
H	0	0	0	0	0	12(100%)

Supplementary Table 6 The six pollen-climate groups based on HCA and MDS analysis.

Group	Climatic Features	Pollen Taxa
G1	Warm and Dry	Artemisia, Chenopodiaceae, Gramineae
G2	Dry	Nitraria, Ephedra
G3	Warm	Compositae, Cupressaceae
G4	Cold and Humid	Picea
G5	Cold	Betula
G6	Slightly Humid	Rhamnaceae, Pinus

Supplementary Table 7 The pollen-precipitation index P and modern precipitation R-P ranges of the six vegetation types.

Vegetation type	Elevation range	P	R-P range
C	3200-3600	11.45	/
D	3000-3200	29.93	383.69-416.09
E	2000-2600	27.55	221.69-318.89
F	1500-2000	6.07	140.69-221.69
G	1300-1500	2.96	108.29-140.69
H	1100-1300	1.69	75.89-108.29

Supplementary Table 8 The corresponding depth of time periods A, B and C in HX, HS, SJC, QTH01, QTH02, QTL–03, SKJ and JTL sections.

Section	Depth of time period A	Depth of time period B	Depth of time period C
HX	210-324 cm	130-210 cm	0-130 cm
HS	250-620 cm	10-250 cm	0-10 cm
SJC	280-700 cm	150-280 cm	0-150 cm
QTH01	430-640 cm	225-430 cm	0-225 cm
QTH02	370-640 cm	270-365 cm	0-270 cm
QTL-03	/	165-380 cm	0-165 cm
SKJ	170-350 cm	100-170 cm	0-100 cm
JTL	140-300 cm	40-140 cm	0-40 cm
